# Maternal and Neonatal Outcomes Among Women with And Without Severe Acute Respiratory Syndrome Corona Virus-2 Infection: a Retrospective Analytical Study

**DOI:** 10.34763/jmotherandchild.20212502.d-21-00021

**Published:** 2022-04-01

**Authors:** Pratyasha Peepal, Tanushree Sandipta Rath, Saurav Nayak, Sujata Pendyala

**Affiliations:** 1 Department of Obstetrics and Gynecology, IMS & SUM Hospital, Siksha O Anusandhan (Deemed to be) University, Bhubaneswar - 751003, Odisha, India; 2 Department of Biochemistry, All India Institute of Medical Sciences, Bhubaneswar - 751019, Odisha, India

**Keywords:** Asymptomatic, caesarean section, COVID-19, neonate, primigravida, SARS CoV-2, vertical transmission

## Abstract

**Background:**

Corona virus disease (COVID-19) is an infectious disease caused by the novel corona virus known as severe acute respiratory syndrome corona virus 2 (SARS Cov-2). Physiological changes occurring during pregnancy can have a positive or negative effect on the disease progression. The objective of the study was to evaluate the maternal and neonatal outcomes in pregnant women with COVID-19 compared to pregnant women without COVID-19 and to determine its influence on the healthcare system.

**Material and methods:**

This was a retrospective analytical study conducted at a tertiary care hospital in Odisha, India, over 3 months, from 1 September 2020 until 30 November 2020. Results were compared in both groups.

**Results:**

Three hundred and three (303) women delivered, out of whom 92 were COVID-19 positive. Incidence of COVID-19 positivity was 30.3% with 93.47% asymptomatic patients. The majority of the patients were 26–35years of age. Average gestational age at delivery for both groups was 37–40 weeks. COVID-19 positivity was seen more in primigravidas than in multigravidas. Comorbidities such as GDM/type 2 DM, PIH, PROM, APH and jaundice were similar in both groups and statistically non-significant, whereas association of anaemia and hypothyroidism were statistically significant (p<0.05) in the positive group. A single maternal death was reported in the positive group. There was an increase in Caesarean section (p=0.002) with higher incidence of preterm births and lowbirth weights in the positive group. Only 3 babies tested positive for COVID-19, so vertical transmission probability was low. Overall, all babies were healthy and the majority of women were discharged without any complications.

**Conclusion:**

There was no significant effect of the infection on maternal and fetal outcomes, but further studies and long-term follow-up is needed to look for any delayed effects on the babies and mothers.

## Introduction

Corona virus disease (COVID-19) is an infectious disease caused by a newly discovered coronavirus known as severe acute respiratory syndrome coronavirus 2 (SARS CoV-2) [1,2]. The invisible virus has engulfed the whole world, causing the collapse of the human health care system. Many studies are underway to find out the exact origin of the deadly virus that caused the COVID-19 pandemic and how the virus made its way from animals to humans, wreaking havoc in the entire world and affecting more than 134 million people and causing more than 2.9 million deaths [3,4,5]. Data on illness and pregnancy-related outcomes associated with other strains of coronavirus such as severe acute respiratory syndrome (SARS CoV) and the Middle East respiratory syndrome (MERS-CoV) are available, but information is sparse on COVID-19 and its impact on pregnancy [6,7,8]. But pregnancy being a highly vulnerable condition, it might carry the risk of adverse obstetrical and neonatal consequences from the viral disease. The United States Centre for Disease Control (CDC) has stated that pregnant females are at equal risk as females who are not pregnant and some researchers believe that due to relative immunesuppression during pregnancy, excessive inflammation caused by COVID-19 might not cause severe respiratory complications [9,10]. But some are of the opinion that due to changes in the immune system and shift from cellular to humoral immunity, pregnancy puts women at a greater risk of viral pneumonia [11]. Though pregnant women with COVID-19 are less likely to exhibit symptoms such as fever, breathlessness or myalgia, they are at an increased risk of requiring treatment in the intensive care unit (ICU) and may require more invasive ventilation when compared to their non-pregnant counterparts. They are also at an increased risk of preterm delivery and maternal morbidity and mortality[12].Studies conducted during the SARS (severe acute respiratory syndrome) and MERS (middle east respiratory syndrome) outbreaks have found higher incidences of adverse perinatal outcomes such as DIC (disseminated intravascular coagulation), abruption, abortion, preterm births, fetal distress, intrauterine growth retardation and NICU (neonatal intensive care unit) admission[13].Consequently, the deadly COVID-19 pandemic invoked anticipations, apprehensions, miscalculations and many negatives in the minds of obstetricians when the pandemic hit the world.

## Objective of the study

This study was carried out to evaluate the maternal and neonatal outcomes in pregnant women with COVID-19 as compared to pregnant women without COVID-19, and to highlight its effect on our healthcare services.

## Material and methods

A retrospective analytical study was done in the department of Obstetrics and Gynecology, at a tertiary care institute of Odisha, having a dedicated COVID-19 hospital where patients from different parts of the state were referred for treatment and delivery. A total of 303 pregnant women were included in the study, conducted over a period of 3 months from 1 September 2020 until 30 November 2020.It included 92 pregnant women who were diagnosed as COVID-19 positive based on RTPCR reports. Controls were 211 pregnant women who tested COVID-19 negative by RTPCR during the same time frame. All antenatal admissions, whether emergency or routine admissions to the labour room, were subjected to the RTPCR test for COVID-19. Until the reports were obtained, the patients were treated in the isolation ward in the O&G Department. If negative reports were obtained, then they were shifted to the ward or labour room for further treatment and delivery. Any such patients who delivered were included in our study. If a patient tested RTPCR positive, then she was shifted to the COVID-19 hospital and her treatment was further taken up from there. Such patients were also included in our study.

If the delivery was imminent before obtaining the RTPCR report, then the patient was presumed to be a positive case and was delivered or operated in the isolation labour room, according to COVID-19 guidelines, taking all safety precautions. The patient was then followed up postpartum with her RTPCR report as soon as possible and proceeded for further treatment accordingly. Such patients were not included in this study.

Other patients who were referred to the COVID-19 hospital on the basis of prior positive test reports from other parts of the state were directly admitted there and underwent required treatment. They were included in our study.

Details about the patients included age, obstetric index, gestational age, systemic comorbidities, mode of termination and complications during pregnancy as well as in the postpartum period. The mean birth weights of infants, APGAR score at 1 minute and 5 minutes, and rates of livebirth, intrauterine fetal death, stillbirth and various neonatal complications were recorded and used for analysis of the neonatal outcomes. All the newborns were subjected to routine COVID-19 RTPCR within 24 hours of birth. All the parameters were analysed using the Statistical Package for the Social Sciences version 25 software. The Chi-square test was used to compare the variables in both the groups. P value less than 0.05 was considered significant.

## Results

### Incidence of COVID-19 infection in pregnancy

**Fig. 1:**
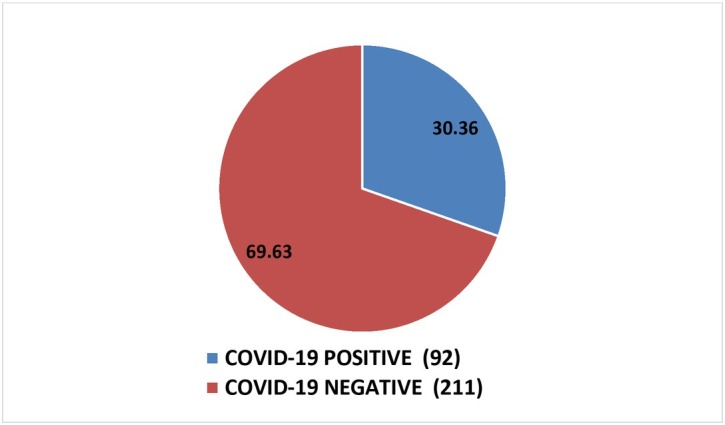
Incidence of COVID-19 infection in pregnancy

Figure 1 shows the incidence of COVID-19 infection in pregnancy among the study population. A total of 303 pregnant females delivered during the study period, out of which 92 women tested COVID-19 positive and the remaining 211 were COVID-19 negative. Thus the incidence of COVID-19 infection in pregnancy was 30.36%.

## Clinical features in COVID-19 positive mothers

The majority of the COVID-19 positive mothers (93.47%) were asymptomatic and incidentally diagnosed as COVID-19 positive based on the routine hospital protocol. Out of the 92 patients, 3 presented with fever, 2 with cough and 1patient with fatigue and shortness of breath.

## Distribution of patients included in the study according to age, parity and gestational age at delivery

Table 1 shows the age distribution of women in both the groups of the study. The majority of the patients in both groups were 26–35 years of age and the mean age was found to be 27.5 years. However, the number of women delivering in the younger spectrum of the reproductive age (below 20 years), which is otherwise considered a high-risk pregnancy, was significantly higher in the COVID-19 positive group (9.7%), as compared to the COVID-19 negative group (0.47%) (p value<0.001). Twenty-six (26) women (28.2%) in the positive group and 78 women (36.96%) in the negative group delivered at an advanced maternal age (above 35years).

**Table 1:** Age-wise distribution of patients in COVID-19 positive and negative groups

Age (Years)	Covid-19 positive n(%)	Covid-19 negative n(%)	Total	p value
<=20	9 (9.78)	1 (0.47)	10	**<0.001**
21-25	21(22.82)	36(17.06)	57	
26-35	36(39.13)	96(45.49)	132	
>35	26(28.26)	78(36.96)	104	
TOTAL	92	211	303	

**Fig. 2:**
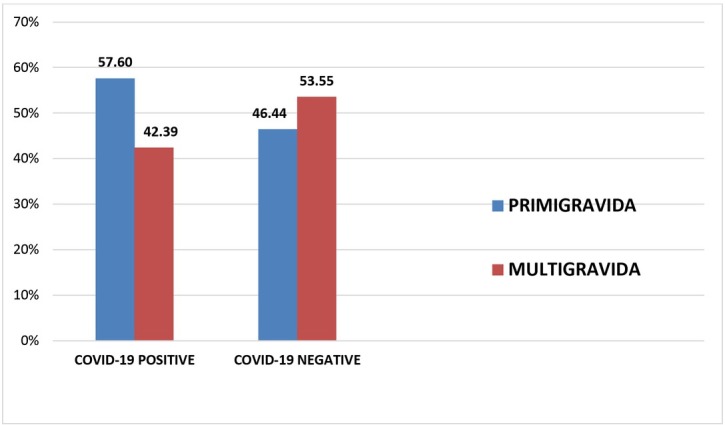
Comparison of parity of women in the study population

Figure 2 shows the parity of women included in the study. Primigravidas were in the majority in the COVID-19 positive group, as compared to multigravidas (57.6% vs 42.39%). In contrast, there was higher number of multigravidas in the COVID-19 negative group as compared to primigravidas (53.55% vs 46.44%). Thus COVID-19 positivity was seen predominantly in the primigravida women, more than that in the multigravida group. However, this association was not statistically significant (p=0.081).

Table 2 shows the gestational age at the time of delivery in both the COVID-19 positive and negative groups. The gestational age at delivery for both groups was between 37–40 weeks. In both the groups most women delivered at term. Nine (9) (9.78%) women in the positive group and 18 (8.53%) in the control group had preterm delivery before 34 weeks of gestation, while 2 women in the positive group had post–mature births (after 42 weeks). But the association was not significant (p value=0.427).

**Table 2:** Gestational age at delivery

Gestational Age (In Weeks)	Covid-19 positive n (%)	Covid-19 negative n (%)	Total	p value
<34	9 (9.78)	18 (8.53)	27	0.427
34-36.6	7(7.60)	30(14.21)	37	
37-41.6	74(80.43)	163(77.25)	237	
>42	2(2.17)	0(0.00)	2	
TOTAL	92	211	303	

## Maternal comorbidities and complications

Table 3 shows the maternal comorbidities associated with antenatal patients in both the groups. Out of the 92 COVID-19 patients,48 women (52.17%) had anaemia (haemoglobin less than 11gm/dl) as compared to 75 out of 211 (35.5%) women in the negative group (p value=0.001 which was statistically significant). There were 6 patients with hypothyroidism in the positive group compared to 37 in the negative group (p value was significant=0.012).Other maternal risk factors such as Type 2 DM/GDM, PIH, PROM/PPROM, Rh negative pregnancy, APH and intra-hepatic cholestasis of pregnancy were present similarly in both groups and the association was statistically non-significant (p>0.05).A single maternal death occurred in the COVID-19 positive group, a 25-year-old primigravida with gestational hypertension who delivered at 34 weeks of gestation and succumbed to the infection two weeks post-delivery.

**Table 3:** Maternal co-morbidities and complications

PARAMETERS	Covid-19 positive n (%)	Covid-19 negative n (%)	p value
1.Hypothyroidism	6 (6.52)	37 (17.53)	**0.012**
2.GDM/Type 2 DM	2 (2.17)	9 (4.26)	0.514
3.PIH	2 (2.17)	16 (7.58)	0.069
4.PPROM/PROM	9(9.78)	28(13.27)	0.450
5.APLA Syndrome	0(0)	4(1.89)	0.318
6.Rh negative pregnancy	2(0)	5(2.36)	1.000
7placentae) .APH(placenta previa/abruption	0(0)	5(2.36)	0.328
8anaemia/.Hemoglobinopathies(Thalassemia) Sickle cell	0(0)	2(0.94)	1.000
9.Cholestasis of pregnancy	0(0)	3(1.42)	0.556
10.Anaemia(Hemoglobin<11gm/dl)	48(52.17)	75(35.54)	**0.001**

GDM–gestational diabetes mellitus, DM–diabetes mellitus, PIH–pregnancy-induced hypertension, PPROM–preterm prelabour rupture of membranes, PROM–prelabour rupture of membranes, APLA–antiphospholipid antibodies, APH–antepartum haemorrhage

## Mode of delivery

**Fig. 3:**
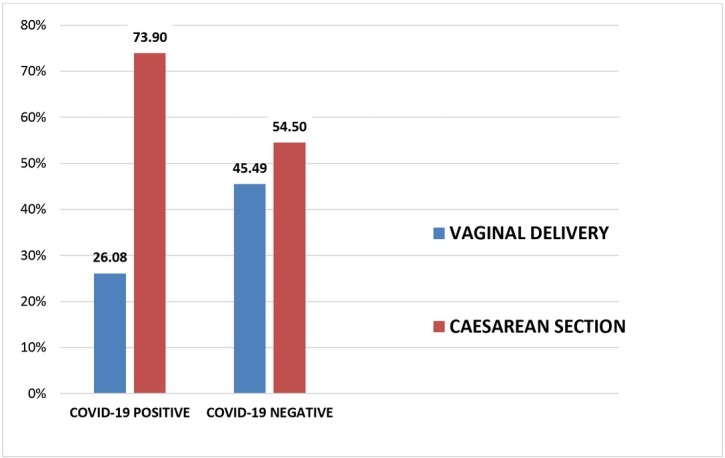
Mode of delivery

Figure 3 shows the mode of delivery in both the groups of patients. In the COVID-19 positive group, 24 patients (26.08%) had a vaginal delivery and 68 patients (73.9%) were delivered by C-section. Similarly, in the COVID-19 negative group, 96 women (45.49%) delivered vaginally whereas 115 women (54.5%) had a C-section. Thus the number of patients who delivered by Caesarean section was higher in the cases than controls and this association was found to be statistically significant (p value=0.002).Those patients who were received in the COVID-19 labour room in active labour, with a COVID-19 positive report, were given a trial of vaginal delivery, but those in latent labour or with obstetrical complications had a C-section without any standard indications, in view of the pandemic scenario, with the aim to reduce the risk of transmission of infection to health care workers attending the patient for long hours and considering controversies regarding vertical transmission.

## Indications of LSCS

Table 4 shows the various indications of LSCS in both groups of patients. The indications such as previous LSCS, fetal distress, PROM, IUGR, breech presentation, APH, multiple pregnancy and doppler changes in USG had almost similar rates in both the groups and was not statistically significant (p>0.05).Elective LSCS without any standard obstetric indication was performed in 14 patients (15.2%) in the positive group as compared to 10 (4.7%) in the negative group (p value statistically significant, p=0.002) as per the hospital protocol due to the emergency pandemic situation.

**Table 4:** Indications of LSCS performed in the study population

Indication	COVID-19 positive n (%)	COVID-19 negative n (%)	p-Value
Previous LSCS	18(19.56)	42(19.90)	0.540
Fetal distress	12(13)	20(9.47)	0.231
Elective LSCS	14(15.21)	10(4.73)	**0.002**
Oligohydramnios	3(3.26)	6(2.84)	0.549
Breech presentation	1(1.08)	7(3.31)	0.443
Multiple pregnancy	3(3.26)	7(3.31)	1.000
IUGR	0(0)	8(3.79)	0.111
APH	0(0)	5(2.36)	0.328
Doppler USG changes in	0(0)	2(0.94)	1.000

## Comparison of the neonatal outcomes and NICU admissions

**Fig. 4:**
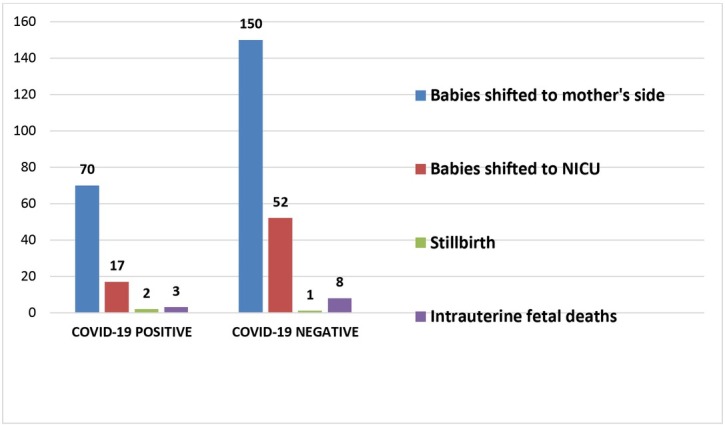
Neonatal outcomes in babies born to both groups of women

Figure 4 shows 70 babies (76%) in the positive group and almost a similar percentage of babies (71%), that is, 150 babies, in the negative group were healthy and transferred to the mother’s side after delivery. In the COVID-19 positive group, there were 2 cases of stillbirth, 3 cases of intrauterine fetal deaths and 17 babies were admitted to NICU due to various neonatal complications such as respiratory distress, prematurity or neonatal jaundice. There were reported cases of 1stillborn baby, 8 cases of IUFD and 52 NICU admissions in the COVID-19 negative group. Only 3 newborns tested positive for COVID-19 in the COVID-19 positive mothers’ group and among them 2 were asymptomatic and the third neonate had respiratory distress. The rest of all newborns with respiratory distress who were admitted to NICU had tested negative for COVID-19, and had the condition due to other causes such as prematurity or meconium aspiration.

**Fig. 5:**
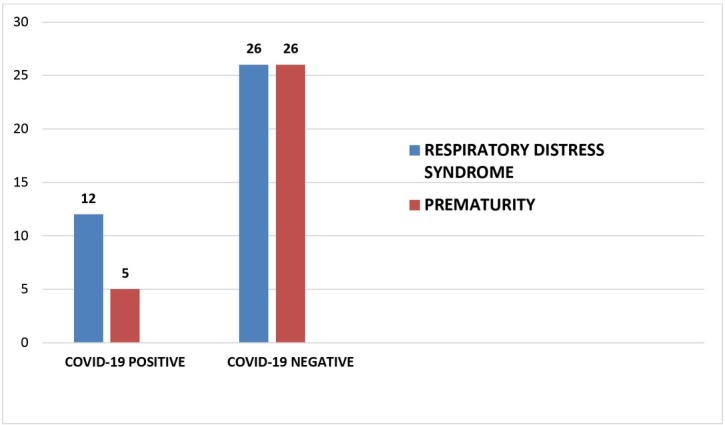
Indications of NICU admission

Figure 5 shows the common indications of NICU admissions in babies born to both the groups of women. Five babies out of 92 (5.43%) born to the COVID-19 positive mothers had NICU admission due to prematurity as opposed to 26 babies out of 211 born (12.32%) in the control group (**p value significant=0.048**).Complications such as respiratory distress and neonatal jaundice were noted in 12 babies (13.04%) in the positive group and 26 babies (12.32%) in the negative group (p value not significant).

## Comparison of birth weights in both the groups

Table 5 shows the distribution of birth weights in babies born to COVID-19 positive and negative mothers. Percentage of babies with very low birth weight (<1.5kg) was 7.6% in the positive group and 6.6% in the negative group and percentage of high birth weight babies (>3.5kg) was found to be 21.7% in the positive group and 9% in the negative group. So extremes of birth weight were more prevalent in the positive group than in the negative group (p value was statistically significant =0.001).

**Table 5:** Comparison of birth weights in both the groups of women

Birth Weight (In Kg)	Covid-19 positive n (%)	Covid-19 negative n (%)	Total	p value
>3.5	20(21.73)	19(9.00)	39	**0.001**
3-3.4	28(30.43)	53(25.11)	81	
2.5-2.9	28(30.43)	67(31.75)	95	
2-2.4	3(3.26)	41(19.43)	44	
1.5-1.9	6(6.52)	17(8.05)	23	
<1.5	7(7.60)	14(6.63)	21	
TOTAL	92	211	303	

## Comparison of APGAR score at 1 minute and 5 minutes

The APGAR score at 1minute [Figure 6(a)] and 5 minutes [Figure 6(b)] was normal in most of the neonates in both the groups. Very few newborn babies were moderately depressed at birth (APGAR 4-6) and severely depressed (APGAR 0-3), indicating that most of the neonates were healthy, whether born to COVID-19 positive or negative mothers.

**Fig. 6(a):**
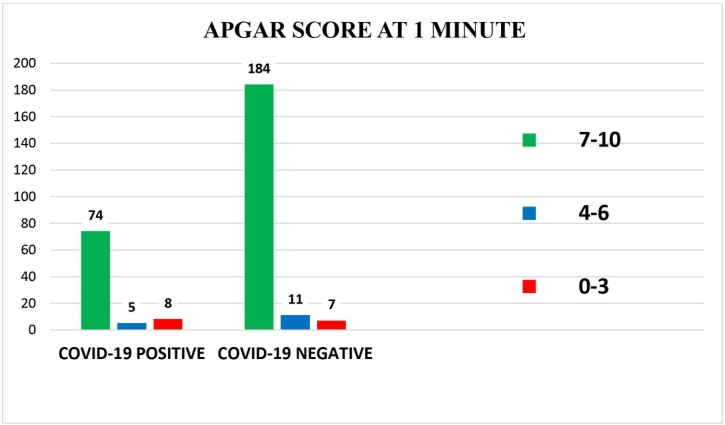
Comparison of APGAR scores at 1minute (COVID-19 Positive live birth =87 COVID-19 Negative live birth = 202)

**Fig. 6(b):**
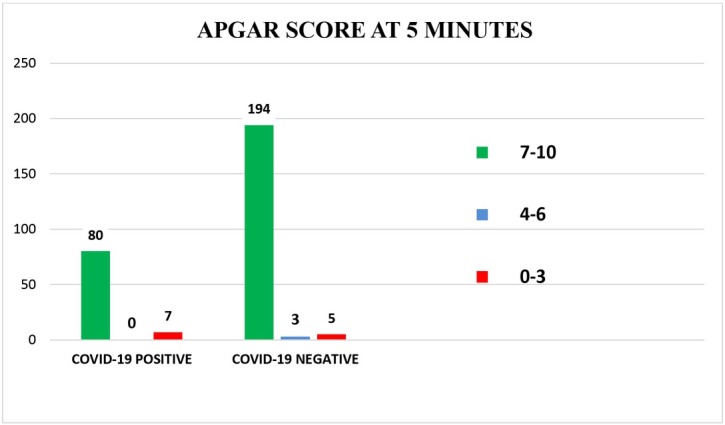
Comparison of APGAR scores at 5 minutes (COVID-19 Positive live birth =87 COVID-19 Negative live birth = 202)

## Discussion

Corona virus may present with an array of clinical features ranging from upper respiratory tract symptoms to muscle pain to gastrointestinal symptoms such as abdominal pain and diarrhoea and sometimes the severe form of the illness in the form of pneumonia or multi-organ dysfunction, may require intensive care [14].

The incidence of COVID-19 infection in pregnancy in this study was 30.36 % and the majority of these (93.47%) were asymptomatic. Others presented with mild symptoms such as cough, fever, weakness and myalgia. Perez et al., through their retrospective study on perinatal consequences of SARS-CoV-2 infection, had similar results. In their study, the prevalence of COVID-19 was 29%, similar to our study, and among them, 86% of the COVID-19 positive patients were asymptomatic while only 9 patients presented with mild infection and 1 patient presented with moderate form of the infection[15].Nan Yu et al. performed a retrospective study for evaluating obstetric and neonatal outcomes and concluded that the perinatal outcomes in patients infected with the virus in the last trimester was quite good with clinical features similar to their non-pregnant counterparts. However, active and intensive management is the key for best results, due to absence of concrete data in the present scenario [16].

Zohra et al. reviewed COVID-19 severity and perinatal outcomes in confirmed COVID-19 pregnant women and reported that the average maternal age was 30.9 years, similar to our study (27.5 years). But in their study, women with severe forms of the infection were 3.7 years older and the risk of getting severe COVID-19 was 1.5 times more if the maternal age was more than 35 years [17].

In our study, COVID-19 positivity was found more in the primigravida women, which is in contrast to the study of Singh et al., who studied the maternal and neonatal outcomes of COVID-19 in pregnancy and evaluated 132 pregnant women and found slightly increased prevalence of COVID-19 positivity among multigravidas [18]. Similarly, Nayak et al., through their preliminary study of coronavirus in pregnancy, found that most of the patients were in the age bracket of 21–35 years and a greater number of multigravidas tested COVID-19 positive [19].

In our study the majority of women in both the positive and negative groups delivered at term. Out of the 92 pregnant women with COVID-19 infection, 16 women (17.39%) had preterm deliveries before 37 completed weeks. Daniele et al., through their meta-analysis on outcome of coronavirus spectrum infections, reported that preterm birth was the most common adverse obstetrical outcome along with higher rates of pre-eclampsia, Caesarean sections and perinatal deaths in COVID-19 positive women, but clinical evidence of vertical transmission is lacking, which is similar to our study [20]. Similarly, Chi et al. summarised that pregnant females were mildly ill, with mortality lower than the overall COVID-19 mortality and Caesarean section was more common, along with premature delivery [21].

In the current study, maternal comorbidities such as gestational diabetes mellitus/Type 2 diabetes mellitus, pregnancy-induced hypertension, antepartum haemorrhage, and jaundice were comparable in both the groups, whereas anaemia and hypothyroidism were more prevalent in the COVID-19 positive pregnant females, without any additional significant effects on the perinatal outcomes, when compared to their negative counterparts. John et al. reported that presence of comorbidities, advanced maternal age and high BMI (body mass index) serve as some independent risk factors, and preterm births were high in pregnancy with COVID-19 infection as opposed to pregnancy without the infection [22]. Wastnedge et al. explored on the topic of pregnancy and COVID-19 and concluded that the risk factors for severe infection are same as the general population but the risk of severe disease is higher than the general population [23].

Considering the pandemic scenario and controversies with respect to vertical transmission and anticipating the delay in the first stage of labour, the indications for Caesarean section were applied flexibly in our institute and the threshold lowered. In our study, the number of patients who delivered by Caesarean section was higher in the COVID-19 positive group than in the negative group (73.9% vs 54.5%). This is similar to studies conducted by Daniele et al., and Chi et al., who found higher rates of Caesarean section in COVID-19 positive mothers [20, 21].

The most common indications of LSCS in our study were elective LSCS, previous LSCS and fetal distress. Benjamin et al. reviewed pregnancies affected by SARS-CoV-2 to ascertain the frequency of maternal and neonatal complications and concluded that maternal and neonatal mortality and vertical transmission was low, but rate of preterm births was 32% and Caesarean section almost 84.7% , which may be attributed to the geographical practice patterns [24].

The majority of the newborns (76%) in the COVID-19 positive group and 71% in the negative group were healthy and transferred to their mother’s side after delivery. There were 17 NICU admissions among newborns in the COVID-19 positive mothers’ group (5 due to prematurity and 12 due to respiratory distress syndrome). Considering the fact that only 3 newborns out of 92 in the COVID-19 positive mothers’ group tested positive for COVID-19, it is assumed that the risk of vertical transmission was apparently low. Studies by Daniele et al. and Benjamin et al. also agree with this rate[20,24].

Among the 3 neonates testing positive for COVID-19, 2 were asymptomatic and the third neonate had respiratory distress. The rest of all newborns with respiratory distress who were admitted to the NICU had tested negative for COVID-19, and had the condition due to other causes such as prematurity or meconium aspiration. Therefore respiratory distress due to COVID-19 infection was likely in only 1 neonate who tested positive.

In our study, very low birth weight babies (<1.5kg) and big babies (birth weight>3.5kg) were more in the positive group than in the negative group and the APGAR scores at 1minute and 5 minutes were normal in most of the neonates in both the groups, in contrast to the findings of Ciapponi et al., who found from their systematic review that COVID-19 carries an increased risk of adverse pregnancy and birth outcomes, although with low risk of congenital transmission [25]. Rafael et al. also differ from our findings on the perinatal outcomes and found that although the virus had a relatively benign course in pregnant women, the neonates appeared to be affected more; proof of vertical transmission was lacking [20, 26]. Lemi et al. also concluded on the low probability of vertical transmission of COVID-19 and stated that there are no sufficient data in the context of abstaining from breast feeding, Caesarean section or mother and neonate separation to avoid vertical transmission. Hence more research with a good sample size of breast milk, amniotic fluid, cord blood and placenta is needed to support the possibility of vertical transmission [27].

## Conclusion

The scientists and health care professionals are working around the clock on the epidemiology, vaccines and management protocols for the novel coronavirus. Still there is a paucity of data on maternal and fetal outcomes in pregnant females with COVID-19 infection. Results of our study suggest that:

- The virus has negligible impact on the mother and fetus. Asymptomatic COVID-19 in pregnancy was common but might have some unknown clinical importance.
- The majority of the patients in both the groups delivered at term and COVID-19 positivity was seen more in primigravidas than multigravidas.
- Comorbidities such as GDM/type2 DM, PIH, PPROM, APH and jaundice were similar in both the groups and the association was statistically non-significant.
- Anaemia and hypothyroidism were more prevalent in COVID-19 positive pregnant females and the association was statistically significant, although with no major difference in perinatal outcomes when compared to the COVID-19 negative group. A single maternal death was reported in the positive group, a 25-year-old primigravida with gestational hypertension, who succumbed two weeks postpartum after delivery at 34 weeks, due to COVID-19 pneumonia.
- There was an increase in the LSCS rates, higher incidence of preterm births and low birth weights in the positive group.
- Only 3 babies tested positive for COVID-19 infection, so vertical transmission is a likelihood, but the probability is low.

But doubts on delayed effects on babies and mothers still remain, and are far from being allayed. The studies on COVID-19–related obstetrical and neonatal outcomes that would enable one to draw unprejudiced conclusions with respect to its specific complications during pregnancy are inadequate. But extensive research is currently underway, with regular updates of data in this field. There is a huge gap between anticipation and observation and between rhetoric and reality. So, the need of the hour is good antenatal and perinatal care and long-term follow-up of the patients to get a clearer picture of effects of COVID-19 infection in pregnancy, labour and the neonates.
